# Strauss‐Defined LBBB Identifies Patients With Improved Outcomes After CRT‐D: A Comparative Cohort Study

**DOI:** 10.1002/clc.70408

**Published:** 2026-07-06

**Authors:** Athanasios Saplaouras, Konstantinos Pamporis, Panagiotis Mililis, Stavroula Koskina, Athanasios Makris, Sokratis Oikonomou, Theodoros Efremidis, Athena Batsouli, Ourania Kariki, George Bazoukis, Sotirios Xydonas, Stylianos Dragasis, Theodoros Karamitsos, Christodoulos Papadopoulos, Nikolaos Fragakis, Michael Efremidis, Konstantinos P. Letsas

**Affiliations:** ^1^ Department of Cardiology Onassis Hospital Athens Greece; ^2^ Aristotle University of Thessaloniki Thessaloniki Greece; ^3^ Department of Cardiology Evaggelismos Hospital Athens Greece; ^4^ Department of Cardiology Larnaca General Hospital Larnaca Cyprus

**Keywords:** cardiac resynchronization therapy, clinical outcomes, electrocardiography, heart failure, left bundle branch block, Strauss criteria

## Abstract

**Background:**

Left bundle branch block (LBBB) is a well‐established predictor of response to cardiac resynchronization therapy (CRT) in heart failure with reduced ejection fraction. However, several electrocardiographic definitions are used, and their relative significance remains unclear.

**Hypothesis:**

Stricter electrocardiographic definitions of LBBB are associated with improved response to CRT and more favorable clinical outcomes.

**Methods:**

We conducted a retrospective single‐center cohort study including 109 patients who underwent CRT‐defibrillator implantation. Baseline electrocardiograms were classified according to six commonly used LBBB definitions. The primary endpoint was echocardiographic response at 6 months, defined as a ≥ 10% absolute increase in left ventricular ejection fraction or a ≥ 15% reduction in left ventricular end‐systolic volume. Secondary endpoints included heart failure hospitalization and all‐cause mortality. Multivariable logistic, Cox, and negative binomial regression models were used, with odds ratios (OR), hazard ratios (HR), incidence rate ratios (IRR), and 95% confidence intervals (CI).

**Results:**

Strauss‐defined LBBB was independently associated with echocardiographic response (adjusted OR, 7.47; 95% CI, 2.52–25.0; *p* < 0.001) and showed the highest discriminative performance (area under the curve, 0.728; 95% CI, 0.643–0.814). Strauss was also associated with lower heart failure hospitalization rates (adjusted IRR, 0.30; 95% CI, 0.13–0.64; *p* = 0.002) and reduced mortality (adjusted HR, 0.14; 95% CI, 0.02–0.99; *p* = 0.049). Median follow‐up was 30 months (IQR 14–60).

**Conclusions:**

Different LBBB definitions are associated with differences in CRT response and outcomes. Stricter criteria appear to better identify patients more likely to respond, with more favorable outcomes. These findings may help refine patient selection for CRT.

## Summary

- Six LBBB definitions compared in 109 CRT‐D patients.
- Strauss best identified echocardiographic responders (aOR 7.47; AUC 0.728).
- Only Strauss was independently linked to response, hospitalization, and mortality.
- Strauss uniquely paired strong resynchronization (QRS narrowing) with response.
- Stricter ECG criteria may improve CRT patient selection.

## Graphical abstract

In a retrospective single‐center cohort of 109 patients who underwent CRT‐D implantation, six electrocardiographic definitions of left bundle branch block (LBBB)—Strauss, Marriott, Perrin, ESC 2013, ESC 2021, and WHO/AHA—were compared for their ability to predict outcomes after cardiac resynchronization therapy (CRT). Strauss‐defined LBBB showed the strongest association with echocardiographic response at 6 months (adjusted OR 7.47; AUC 0.728) and was the only definition independently associated with all three endpoints: response, fewer heart failure hospitalizations (adjusted IRR 0.30), and lower all‐cause mortality (adjusted HR 0.14). It was also the only criterion combining marked QRS narrowing in criterion‐positive patients with minimal narrowing in the rest. Stricter, morphology‐based ECG criteria may refine patient selection for CRT.

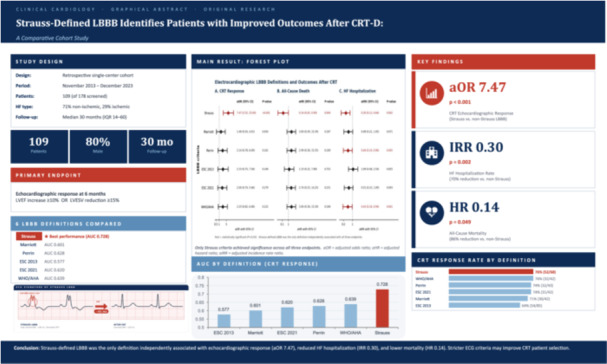

**Abbreviations**
AF: atrial fibrillation
AHA: American Heart Association
AUC: area under the curve
CI: confidence interval
CRT: cardiac resynchronization therapy
CRT‐D: cardiac resynchronization therapy defibrillator
LA: left atrium
Lad: left atrial diameter
LAv: left atrial volume
LAVI: left atrial volume index
LBBB: left bundle branch block
LVEDD: left ventricular end‐diastolic diameter
LVEF: left ventricular ejection fraction
LVESD: left ventricular end‐systolic diameter
LVESV: left ventricular end‐systolic volume
NPV: negative predictive value
NYHA: New York Heart Association Functional Classification
PPV: positive predictive value
SR: sinus rhythm

## 1 Introduction

Cardiac resynchronization therapy (CRT) is recommended for patients with heart failure (HF) with reduced ejection fraction and left bundle branch block (LBBB), aiming to improve symptoms and survival by correcting delayed activation of the left ventricular lateral wall [1]. The presence of LBBB on a 12‐lead electrocardiogram (ECG) is one of the strongest predictors of response to CRT, including reverse remodeling and improved clinical outcomes [2, 3]. However, despite guideline‐based selection, a substantial proportion of patients do not experience significant reverse remodeling after CRT, highlighting the need for more accurate tools to refine patient selection.

One potential determinant of response is the morphology of LBBB on the ECG. Several electrocardiographic definitions are currently used, incorporating different criteria such as QRS duration, septal activation, and QRS morphology. As a result, the same ECG may be classified differently depending on the definition applied, which may influence both patient selection and outcome interpretation [4, 5]. The LBBB pattern itself may reflect different underlying mechanisms, including complete conduction block within the His–Purkinje system, delayed septal conduction despite preserved conduction pathways, or a combination of both [6, 7]. These mechanisms may not respond equally to CRT, suggesting that certain definitions may better identify patients with true electrical desynchrony. Previous studies comparing LBBB definitions have reported heterogeneous findings, likely reflecting differences in study populations, endpoints, and methodology [8, 9, 10, 11, 12]. Therefore, it remains unclear whether contemporary LBBB definitions can reliably identify patients most likely to benefit from CRT. In this context, we aimed to evaluate the association between six established electrocardiographic definitions of LBBB and echocardiographic response, HF hospitalization, and mortality in a single‐center cohort of patients undergoing CRT.

## 2 Methods

### 2.1 Study Population

This was a retrospective observational cohort study based on prospectively collected data from consecutive patients with HF who underwent cardiac resynchronization therapy defibrillator (CRT‐D) implantation. Reporting of the present study followed the Strengthening the Reporting of Observational Studies in Epidemiology (STROBE) guidelines [13].

Patients were screened consecutively between November 2013 and December 2023 at our institution. Inclusion criteria were: (1) ischemic or non‐ischemic HF with left ventricular ejection fraction (LVEF) ≤ 35% despite at least 3 months of optimal medical therapy according to European Society of Cardiology guidelines [1]; (2) presence of LBBB with QRS duration ≥ 130 ms on baseline ECG; (3) ≥ 98% biventricular pacing after implantation in patients with either atrial fibrillation (AF) or sinus rhythm (SR); and (4) comprehensive echocardiographic evaluation before implantation and at 6 months of follow‐up. The present analysis was restricted to patients with LBBB morphology in order to directly compare electrocardiographic LBBB definitions within an LBBB‐selected CRT population.

Exclusion criteria included: (1) device upgrade to CRT; (2) poor echocardiographic windows; (3) absence of LBBB on pre‐implantation ECG; (4) biventricular pacing < 98% due to irreversible causes (e.g., loss of left ventricular capture, lead displacement or fracture, or uncontrolled atrial/ventricular arrhythmias); (5) advanced HF therapies before 6‐month follow‐up; (6) age < 18 years; and (7) pregnancy.

Data on cardiovascular risk factors, comorbidities, functional status, and HF treatment were collected prior to CRT‐D implantation. The study protocol was approved by the hospital ethics committee, and written informed consent was obtained from all participants.

### 2.2 Data Collection

Demographic characteristics, comorbidities, HF etiology, echocardiographic parameters (LVEF, left ventricular end‐systolic volume [LVESV], left ventricular end‐diastolic volume [LVEDV], left ventricular end‐diastolic diameter [LVEDD], left ventricular end‐systolic diameter [LVESD], left atrial diameter and volume [LAd, LAv], and left atrial volume index [LAVI]), electrocardiographic variables (LBBB morphology, PR interval, QRS duration, QT interval), medical therapy, and functional status were recorded at baseline.

Echocardiographic examinations were performed using a GE Vivid 7 system (GE Healthcare, Chalfont St. Giles, UK) during the week prior to CRT implantation and at 6 months. ECGs were digitally scanned and analyzed by two experienced electrophysiologists who were blinded to clinical outcomes at the time of analysis. Disagreements were resolved by consensus, with adjudication by a third experienced electrophysiologist when required. For each LBBB definition, all electrocardiographic criteria had to be fulfilled for classification. Recording speed was 25 mm/s, and sensitivity was 10 mm/mV.

### 2.3 LBBB Definitions

Six established definitions of LBBB were evaluated. Patients were classified according to the following criteria: Strauss‐LBBB, Marriott‐LBBB, Perrin‐LBBB, ESC 2013‐LBBB, ESC 2021‐LBBB, and WHO/AHA‐LBBB. Detailed criteria are presented in Table 1 [14, 15, 16, 17, 18, 19, 20]. All definitions were applied retrospectively to baseline ECGs by two independent reviewers who were blinded to clinical outcomes. Decisions regarding CRT implantation were based on contemporary guideline criteria at the time of implantation and were not influenced by the study‐specific ECG classification.

**Table 1:** Comparison of electrocardiographic definitions of left bundle branch block.

Criterion	Strauss	Marriott	Perrin	ESC 2013	ESC 2021	WHO/AHA
QRS ≥ 120 ms		+	+	+	+	+
QRS ≥ 130 ms (F); QRS ≥ 140 ms (M)	+					
R peak time > 60 ms V5/V6		+	+			+
rS or QS in V1/V2 (with R peak time < 60 ms)	+	+	+	+	+	+
Monophasic R in I		+	+			+
Monophasic R in V6		+				
QRS notching or slurring in ≥ 1 leads between I, aVL, V5, V6			+	+		+
Mid‐QRS notching or slurring in ≥ 2 leads between I, aVL, V1, V2, V5, and V6	+				+	
No q wave in I, V5, V6		+	+			+
No q wave in V5, V6				+		
Monophasic R or qR in aVL			+			+
Generally, the ST segment is slightly opposed to the QRS polarity.					+	
No Q waves ≥ 1 mm in aVL and no Rwave ≥ 1 mm in V1			+			
QS or rS in V1 with ST slightly elevated and positive asymmetrical T wave and unique R wave in V6 with negative asymmetric T wave. When the QRS is less than 140 ms the T wave in V6 may be positive					+	
Exclusive R wave in I and aVL, often with a negative asymmetrical T wave, slight ST depression, and usually QS in aVR with positive T wave					+	
The QRS axis is variable					+	

*Note:* F, female, M, male, a, Mid‐QRS notching or slurring in ≥ 2 leads between I, aVL, V1, V2, V5, and V6.

### 2.4 Follow‐Up

The primary endpoint of the study was echocardiographic response at 6 months following CRT implantation. At the 6‐month visit, patients underwent clinical assessment, electrocardiography, echocardiography, treatment optimization, and device interrogation [21]. Clinical outcomes, including HF hospitalization and all‐cause mortality, were collected longitudinally from implantation until last contact or death. Hospitalization for HF was defined as admission to a healthcare facility for ≥ 24 h due to HF decompensation requiring medical or interventional treatment [22]. Deaths were verified through review of medical records and, when necessary, telephone contact with first‐degree relatives.

### 2.5 Statistical Analysis

Statistical analyses were performed using R software (version 4.4.2). Categorical variables are presented as frequencies and percentages, while continuous variables are expressed as mean and standard deviation (SD) when normally distributed or median with interquartile range (IQR) otherwise. Normality was assessed visually with Q–Q plots and tested using the Shapiro–Wilk test. No missing data were identified for the analyzed variables.

Associations between LBBB definitions and CRT response were evaluated by: (i) unadjusted diagnostic performance measures (sensitivity, specificity, positive predictive value [PPV], negative predictive value [NPV], and receiver operating characteristic [ROC] curves with area under the curve [AUC]) and (ii) multivariable logistic regression models with odds ratios (OR) and 95% confidence intervals (CI). ROC curves were compared using the DeLong test. Moreover, the level of agreement between different LBBB criteria was evaluated with Gwet's agreement coefficient (AC1) [23], which is a chance‐corrected measure of agreement between classification systems and is selected because of its relative stability in settings with high agreement and unbalanced prevalence [24, 25].

Survival analysis was performed using Kaplan–Meier curves and multivariable Cox proportional hazards models to assess all‐cause mortality, with comparisons by the log‐rank test. Negative binomial regression models were used to evaluate HF hospitalization rates, reported as adjusted incidence rate ratios (IRR). Given the limited number of clinical events, analyses involving hospitalization and mortality outcomes were interpreted as exploratory. The limited number of deaths relative to the number of covariates may have resulted in model instability and overfitting; therefore, the mortality estimates should be considered exploratory. Additional details are provided in Supporting Information S1: Table S1. The proportional hazards assumption was assessed using Schoenfeld residuals and was not violated for the main exposure variables. Given the limited number of mortality events, multivariable models were restricted to clinically relevant covariates to minimize overfitting. Variables included in multivariable models were selected based on clinical relevance and information from prior literature. For all Strauss multivariable models, a sensitivity analysis was performed to identify the strength of unmeasured confounding required to eliminate the significance of the adjusted effect. A two‐sided *p* < 0.05 was considered statistically significant.

## 3 Results

### 3.1 Study Population

**Figure 1:**
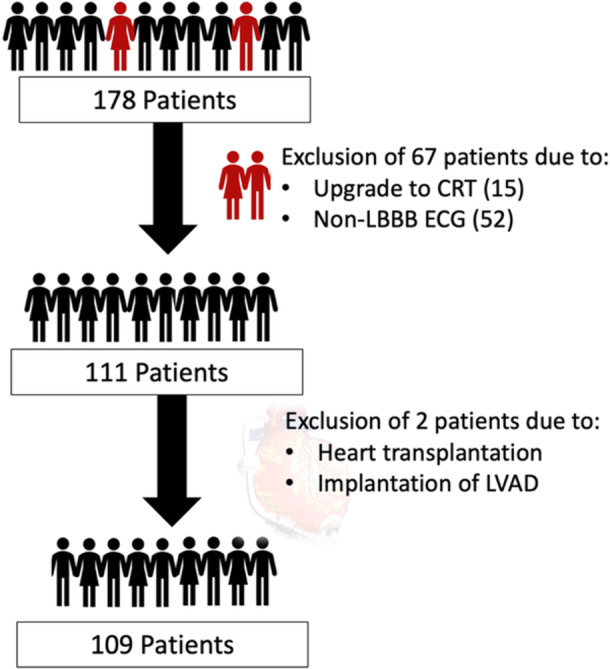
Study flow diagram of patient selection. A total of 178 consecutive patients were screened, of whom 109 were included in the final analysis. CRT, cardiac resynchronization therapy; LBBB, left bundle branch block; LVAD, left ventricular assist device.

A total of 178 consecutive patients with HF who underwent CRT‐D implantation were screened for eligibility. Fifteen patients were excluded because they underwent device upgrade to CRT, and 52 patients were excluded due to the absence of LBBB morphology on baseline ECG. Among the remaining 111 patients, two were excluded during follow‐up because of heart transplantation and left ventricular assist device implantation before the 6‐month assessment, resulting in a final cohort of 109 patients. The study flow, including screening, exclusions, and final cohort selection, is summarized in Figure 1 according to STROBE recommendations.

Baseline characteristics according to CRT response are presented in Table 2, while characteristics stratified by LBBB definitions are summarized in Table 3. Of the 109 patients, 87 (80%) were men, with a mean age of 66 ± 11 years. Non‐ischemic HF was present in 77 patients (71%), and most participants were classified as NYHA functional class III at baseline (86/109, 79%). Median pre‐implantation LVEF was 29% (IQR 25–31), and mean LVESV was 173 ± 66 mL. SR was present in 95/109 patients (87%). In general, key CRT‐related characteristics including baseline QRS, LVEF, LV dimensions, NYHA functional class, and use of guideline‐directed medical therapy, were broadly similar across LBBB groups.

**Table 2:** Characteristics of participants by response to CRT.

Variable	Overall population (*N* = 109)	Responders (*N* = 64)	Non‐responders (*N* = 45)	*p* value
Sex				0.048
Male (%)	87/109 (80)	47/64 (73)	40/45 (89)	
Female (%)	22/109 (20)	17/64 (27)	5/45 (11)	
Age (years)	66 (11)	66 (11)	66 (10)	> 0.9
BMI (kg/m^2^)	26.4 (25.0, 28.7)	26.1 (24.7, 28.9)	26.6 (25.8, 28.3)	0.3
Hypertension (%)	13/109 (12)	6/64 (9.4)	7/45 (16)	0.3
Diabetes mellitus (%)	20/109 (18)	10/64 (16)	10/45 (22)	0.4
Paroxysmal AF (%)	43/109 (39)	20/64 (31)	23/45 (51)	0.037
Thyroid disease (%)	7/109 (6.4)	3/64 (4.7)	4/45 (8.9)	0.4
Dyslipidemia (%)	49/109 (45)	27/64 (42)	22/45 (49)	0.5
HF type				< 0.001
Non‐ischemic (%)	77/109 (71)	53/64 (83)	24/45 (53)	
Ischemic (%)	32/109 (29)	11/64 (17)	21/45 (47)	
NYHA before CRT				0.8
I (%)	0/109 (0)	0/64 (0)	0/45 (0)	
II (%)	20/109 (18)	13/64 (20)	7/45 (16)	
III (%)	86/109 (79)	49/64 (77)	37/45 (82)	
IV (%)	3/109 (2.8)	2/64 (3.1)	1/45 (2.2)	
EF before CRT (%)	29 (25, 31)	29 (25, 30)	30 (25, 35)	0.8
LVESV before CRT (mL)	173 (66)	175 (70)	171 (61)	0.8
LVEDD before CRT (mm)	65 (8)	65 (9)	65 (8)	0.8
LVESD (mm)	52 (48, 62)	53 (48, 61)	52 (48, 63)	0.8
LVEDV before CRT (mL)	250 (79)	250 (81)	249 (76)	> 0.9
LA diameter (mm)	45.3 (5.2)	44.5 (5.2)	46.5 (5.0)	0.019
LA volume (mL)	89 (75, 117)	85 (65, 110)	94 (80, 123)	0.084
LAVI (mL/m^2^)	45 (38, 58)	45 (33, 56)	48 (41, 61)	0.10
Baseline rhythm				0.7
SR (%)	95/109 (87)	55/64 (86)	40/45 (89)	
AF (%)	14/109 (13)	9/64 (14)	5/45 (11)	
QRS duration (ms)	160 (150, 180)	160 (160, 180)	156 (140, 170)	0.011
LBBB Strauss (%)	68/109 (62)	52/64 (81)	16/45 (36)	< 0.001
LBBB Marriott (%)	42/109 (39)	30/64 (47)	12/45 (27)	0.033
LBBB Perrin (%)	43/109 (39)	32/64 (50)	11/45 (24)	0.007
LBBB ESC 2013 (%)	85/109 (78)	54/64 (84)	31/45 (69)	0.055
LBBB ESC 2021 (%)	42/109 (39)	31/64 (48)	11/45 (24)	0.011
LBBB WHO/AHA (%)	42/109 (39)	32/64 (50)	10/45 (22)	0.003
PR segment (ms)	187 (42)	180 (39)	196 (46)	0.067
Fridericia QTc (ms)	470 (440, 495)	470 (440, 500)	470 (440, 490)	0.7
RAAS inhibitor (%)	59/109 (54)	39/64 (61)	20/45 (44)	0.089
B‐blocker (%)	104/109 (95)	62/64 (97)	42/45 (93)	0.6
MRA (%)	93/109 (85)	53/64 (83)	40/45 (89)	0.4
Ivabradine (%)	5/109 (4.6)	1/64 (1.6)	4/45 (8.9)	0.2
Diuretic (%)	98/109 (90)	57/64 (89)	41/45 (91)	> 0.9
Nitrates (%)	6/109 (5.5)	2/64 (3.1)	4/45 (8.9)	0.2
Digoxin (%)	6/109 (5.5)	4/64 (6.3)	2/45 (4.4)	> 0.9
CCB (%)	0/109 (0)	0/64 (0)	0/45 (0)	> 0.9
Anticoagulation (%)	47/109 (43)	24/64 (38)	23/45 (51)	0.2
Antiplatelets (%)	33/109 (30)	14/64 (22)	19/45 (42)	0.023
Antiarrhythmics (%)	35/109 (32)	14/64 (22)	21/45 (47)	0.006
Statins (%)	56/109 (51)	31/64 (48)	25/45 (56)	0.5
SGLT2i (%)	41/109 (38)	24/64 (38)	17/45 (38)	> 0.9
ARNI (%)	37/109 (34)	17/64 (27)	20/45 (44)	0.052
Follow‐up duration (months)	30 (14, 60)	45 (19, 67)	20 (13, 36)	0.002
Target vein				> 0.9
Lateral	90/108 (83%)	54/64 (84%)	36/44 (82%)	
Posterolateral	11/108 (10%)	6/64 (9.4%)	5/44 (11%)	
Anterolateral	4/108 (3.7%)	2/64 (3.1%)	2/44 (4.5%)	
AIV	3/108 (2.8%)	2/64 (3.1%)	1/44 (2.3%)	
EF after CRT (%)	37 (10)	43 (7)	27 (7)	< 0.001
LVESV after CRT (mL)	119 (56)	97 (39)	152 (61)	< 0.001
NYHA after CRT				< 0.001
I (%)	56/109 (51)	53/64 (83)	3/45 (6.7)	
II (%)	17/109 (16)	9/64 (14)	8/45 (18)	
III (%)	30/109 (28)	2/64 (3.1)	28/45 (62)	
IV (%)	6/109 (5.5)	0/64 (0)	6/45 (13)	
Death (%)	15/109 (14)	2/64 (3.1)	13/45 (29)	< 0.001
Hospitalizations				< 0.001
0 (%)	70/109 (64)	57/64 (89)	13/45 (29)	
1 (%)	24/109 (22)	6/64 (9.4)	18/45 (40)	
2 (%)	5/109 (4.6)	1/64 (1.6)	4/45 (8.9)	
3 (%)	5/109 (4.6)	0/64 (0)	5/45 (11)	
4 (%)	2/109 (1.8)	0/64 (0)	2/45 (4.4)	
5 (%)	1/109 (0.9)	0/64 (0)	1/45 (2.2)	
7 (%)	1/109 (0.9)	0/64 (0)	1/45 (2.2)	
8 (%)	1/109 (0.9)	0/64 (0)	1/45 (2.2)	

Abbreviations: AF, atrial fibrillation; AHA, American Heart Association; ARNI, angiotensin receptor neprilysin inhibitor; BMI, body mass index; CRT, cardiac resynchronization therapy; EF, ejection fraction; ESC, European Society of Cardiology; HF, heart failure; LA, left atrium; LAVI, left atrial volume index; LBBB, left bundle branch block; LVEDD, left ventricular end‐diastolic diameter; LVEDV, left ventricular end‐diastolic volume; LVESD, left ventricular end‐systolic diameter; LVESV, left ventricular end‐systolic volume; MRA, mineralocorticoid receptor antagonist; NYHA, New York Heart Association; RAAS, renin–angiotensin−aldosterone system; RWPT, R‐wave peak time; SGLT2i, sodium‐glucose cotransporter type 2 inhibitor; SR, sinus rhythm; WHO, World Health Organization.

**Table 3:** Baseline characteristics according to fulfillment of each LBBB definition.

Variable	LBBB Strauss		LBBB Marriott		LBBB Perrin		LBBB ESC 2013		LBBB ESC 2021		LBBB WHO/AHA	
	Criterion met (*N* = 68)	Criterion not met (*N* = 41)	Criterion met (*N* = 42)	Criterion not met (*N* = 67)	Criterion met (*N* = 43)	Criterion not met (*N* = 66)	Criterion met (*N* = 85)	Criterion not met (*N* = 24)	Criterion met (*N* = 42)	Criterion not met (*N* = 67)	Criterion met (*N* = 42)	Criterion not met (*N* = 67)
Males	53/68 (78)	34/41 (83)	34/42 (81)	53/67 (79)	34/43 (79)	53/66 (80)	66/85 (78)	21/24 (88)	33/42 (79)	54/67 (81)	33/42 (79)	54/67 (81)
Age (years)	67 (10)	64 (12)	65 (11)	67 (11)	65 (11)	67 (11)	66 (11)	67 (11)	65 (11)	67 (11)	65 (11)	67 (11)
BMI (kg/m^2^)	26.2 (25.0, 29.0)	26.6 (25.0, 28.3)	26.2 (24.8, 27.7)	26.5 (25.2, 29.1)	26.1 (24.8, 28.0)	26.5 (25.2, 29.1)	26.3 (25.1, 29.1)	26.5 (24.4, 27.9)	26.1 (24.8, 27.7)	26.5 (25.2, 29.1)	26.1 (24.8, 27.8)	26.5 (25.2, 29.1)
Hypertension (%)	6/68 (8.8)	7/41 (17)	4/42 (9.5)	9/67 (13)	3/43 (7.0)	10/66 (15)	11/85 (13)	2/24 (8.3)	3/42 (7.1)	10/67 (15)	3/42 (7.1)	10/67 (15)
Diabetes mellitus (%)	13/68 (19)	7/41 (17)	7/42 (17)	13/67 (19)	7/43 (16)	13/66 (20)	16/85 (19)	4/24 (17)	6/42 (14)	14/67 (21)	6/42 (14)	14/67 (21)
Paroxysmal AF (%)	27/68 (40)	16/41 (39)	16/42 (38)	27/67 (40)	16/43 (37)	27/66 (41)	37/85 (44)	6/24 (25)	16/42 (38)	27/67 (40)	16/42 (38)	27/67 (40)
Thyroid disease (%)	4/68 (5.9)	3/41 (7.3)	2/42 (4.8)	5/67 (7.5)	2/43 (4.7)	5/66 (7.6)	5/85 (5.9)	2/24 (8.3)	2/42 (4.8)	5/67 (7.5)	2/42 (4.8)	5/67 (7.5)
Dyslipidemia (%)	28/68 (41)	21/41 (51)	18/42 (43)	31/67 (46)	20/43 (47)	29/66 (44)	37/85 (44)	12/24 (50)	19/42 (45)	30/67 (45)	19/42 (45)	30/67 (45)
HF type												
Non‐ischemic (%)	53/68 (78)	24/41 (59)	33/42 (79)	44/67 (66)	33/43 (77)	44/66 (67)	62/85 (73)	15/24 (63)	32/42 (76)	45/67 (67)	33/42 (79)	44/67 (66)
Ischemic (%)	15/68 (22)	17/41 (41)	9/42 (21)	23/67 (34)	10/43 (23)	22/66 (33)	23/85 (27)	9/24 (38)	10/42 (24)	22/67 (33)	9/42 (21)	23/67 (34)
NYHA before CRT												
I (%)	0/68 (0)	0/41 (0)	0/42 (0)	0/67 (0)	0/43 (0)	0/66 (0)	0/85 (0)	0/24 (0)	0/42 (0)	0/67 (0)	0/42 (0)	0/67 (0)
II (%)	14/68 (21)	6/41 (15)	10/42 (24)	10/67 (15)	12/43 (28)	8/66 (12)	19/85 (22)	1/24 (4.2)	11/42 (26)	9/67 (13)	11/42 (26)	9/67 (13)
III (%)	52/68 (76)	34/41 (83)	31/42 (74)	55/67 (82)	30/43 (70)	56/66 (85)	63/85 (74)	23/24 (96)	30/42 (71)	56/67 (84)	30/42 (71)	56/67 (84)
IV (%)	2/68 (2.9)	1/41 (2.4)	1/42 (2.4)	2/67 (3.0)	1/43 (2.3)	2/66 (3.0)	3/85 (3.5)	0/24 (0)	1/42 (2.4)	2/67 (3.0)	1/42 (2.4)	2/67 (3.0)
EF before CRT (%)	30 (25, 34)	25 (23, 30)	30 (25, 31)	28 (25, 33)	29 (25, 31)	30 (25, 35)	30 (25, 35)	27 (24, 30)	30 (25, 31)	28 (25, 33)	30 (25, 31)	29 (25, 35)
LVESV before CRT (mL)	169 (68)	180 (63)	182 (77)	168 (58)	184 (76)	167 (58)	175 (71)	168 (48)	182 (78)	168 (58)	184 (77)	167 (58)
LVEDD before CRT (mm)	64 (9)	66 (8)	65 (8)	65 (9)	66 (8)	64 (9)	65 (8)	65 (9)	65 (7)	64 (9)	66 (8)	64 (9)
LVESD (mm)	52 (48, 60)	56 (50, 63)	54 (49, 60)	52 (47, 62)	54 (50, 63)	52 (47, 62)	52 (48, 60)	53 (49, 63)	54 (50, 60)	52 (47, 62)	55 (50, 63)	52 (47, 62)
LVEDV before CRT (mL)	243 (80)	261 (75)	262 (89)	242 (71)	265 (87)	240 (72)	253 (83)	240 (62)	264 (88)	241 (71)	265 (88)	240 (71)
LA diameter (mm)	44.5 (5.2)	46.8 (5.0)	45.3 (5.4)	45.4 (5.1)	45.0 (5.4)	45.5 (5.2)	45.4 (5.4)	45.3 (4.6)	45.2 (5.6)	45.4 (5.0)	45.1 (5.4)	45.5 (5.1)
LA volume (mL)	85 (68, 117)	90 (80, 110)	85 (65, 110)	90 (80, 120)	85 (65, 110)	90 (80, 119)	85 (69, 117)	94 (82, 110)	84 (65, 110)	90 (80, 120)	84 (65, 110)	90 (80, 120)
LAVI (mL/m^2^)	44 (37, 59)	48 (39, 55)	44 (33, 56)	47 (39, 59)	45 (33, 56)	46 (39, 59)	44 (36, 59)	48 (39, 57)	44 (33, 56)	47 (39, 59)	44 (33, 56)	47 (39, 59)
Baseline rhythm												
SR (%)	60/68 (88)	35/41 (85)	36/42 (86)	59/67 (88)	37/43 (86)	58/66 (88)	73/85 (86)	22/24 (92)	36/42 (86)	59/67 (88)	36/42 (86)	59/67 (88)
AF (%)	8/68 (12)	6/41 (15)	6/42 (14)	8/67 (12)	6/43 (14)	8/66 (12)	12/85 (14)	2/24 (8.3)	6/42 (14)	8/67 (12)	6/42 (14)	8/67 (12)
QRS duration (ms)	160 (160, 180)	150 (135, 160)	160 (160, 180)	156 (140, 170)	160 (160, 180)	155 (140, 170)	160 (150, 180)	153 (150, 178)	160 (160, 180)	156 (140, 170)	163 (160, 180)	154 (140, 170)
RWPT > 60 ms (%)	39/68 (57)	4/41 (9.8)	41/42 (98)	2/67 (3.0)	42/43 (98)	1/66 (1.5)	43/85 (51)	0/24 (0)	41/42 (98)	2/67 (3.0)	42/42 (100)	1/67 (1.5)
PR segment (ms)	187 (45)	185 (37)	179 (42)	191 (42)	183 (45)	189 (41)	185 (43)	192 (42)	180 (42)	190 (42)	180 (42)	190 (42)
Fridericia QTc (ms)	474 (440, 502)	460 (433, 489)	480 (450, 510)	465 (440, 490)	480 (450, 510)	462 (440, 490)	470 (440, 500)	462 (448, 489)	480 (450, 510)	463 (440, 490)	480 (450, 510)	463 (440, 490)
RAAS inhibitor (%)	39/68 (57)	20/41 (49)	27/42 (64)	32/67 (48)	27/43 (63)	32/66 (48)	48/85 (56)	11/24 (46)	28/42 (67)	31/67 (46)	27/42 (64)	32/67 (48)
B‐blocker (%)	66/68 (97)	38/41 (93)	42/42 (100)	62/67 (93)	43/43 (100)	61/66 (92)	82/85 (96)	22/24 (92)	42/42 (100)	62/67 (93)	42/42 (100)	62/67 (93)
MRA (%)	59/68 (87)	34/41 (83)	36/42 (86)	57/67 (85)	37/43 (86)	56/66 (85)	72/85 (85)	21/24 (88)	36/42 (86)	57/67 (85)	36/42 (86)	57/67 (85)
Ivabradine (%)	2/68 (2.9)	3/41 (7.3)	2/42 (4.8)	3/67 (4.5)	2/43 (4.7)	3/66 (4.5)	3/85 (3.5)	2/24 (8.3)	2/42 (4.8)	3/67 (4.5)	2/42 (4.8)	3/67 (4.5)
Diuretic (%)	57/68 (84)	41/41 (100)	35/42 (83)	63/67 (94)	36/43 (84)	62/66 (94)	75/85 (88)	23/24 (96)	35/42 (83)	63/67 (94)	35/42 (83)	63/67 (94)
Nitrates (%)	4/68 (5.9)	2/41 (4.9)	2/42 (4.8)	4/67 (6.0)	2/43 (4.7)	4/66 (6.1)	3/85 (3.5)	3/24 (13)	2/42 (4.8)	4/67 (6.0)	2/42 (4.8)	4/67 (6.0)
Digoxin (%)	1/68 (1.5)	5/41 (12)	0/42 (0)	6/67 (9.0)	0/43 (0)	6/66 (9.1)	5/85 (5.9)	1/24 (4.2)	0/42 (0)	6/67 (9.0)	0/42 (0)	6/67 (9.0)
CCB (%)	0/68 (0)	0/41 (0)	0/41 (0)	0/67 (0)	0/43 (0)	0/66 (0)	0/85 (0)	0/24 (0)	0/42 (0)	0/67 (0)	0/42 (0)	0/67 (0)
Anticoagulation (%)	31/68 (46)	16/41 (39)	17/42 (40)	30/67 (45)	18/43 (42)	29/66 (44)	39/85 (46)	8/24 (33)	17/42 (40)	30/67 (45)	17/42 (40)	30/67 (45)
Antiplatelets	16/68 (24)	17/41 (41)	12/42 (29)	21/67 (31)	12/43 (28)	21/66 (32)	24/85 (28)	9/24 (38)	11/42 (26)	22/67 (33)	11/42 (26)	22/67 (33)
Antiarrhythmics	19/68 (28)	16/41 (39)	16/42 (38)	19/67 (28)	16/43 (37)	19/66 (29)	26/85 (31)	9/24 (38)	15/42 (36)	20/67 (30)	15/42 (36)	20/67 (30)
Statins (%)	32/68 (47)	24/41 (59)	21/42 (50)	35/67 (52)	21/43 (49)	35/66 (53)	44/85 (52)	12/24 (50)	20/42 (48)	36/67 (54)	20/42 (48)	36/67 (54)
SGLT2i (%)	24/68 (35)	17/41 (41)	12/42 (29)	29/67 (43)	13/43 (30)	28/66 (42)	30/85 (35)	11/24 (46)	12/42 (29)	29/67 (43)	12/42 (29)	29/67 (43)
ARNI (%)	20/68 (29)	17/41 (41)	10/42 (24)	27/67 (40)	11/43 (26)	26/66 (39)	26/85 (31)	11/24 (46)	10/42 (24)	27/67 (40)	10/42 (24)	27/67 (40)
Follow‐up duration (months)	33 (14, 61)	24 (15, 48)	32 (16, 69)	26 (14, 55)	32 (17, 69)	25 (14, 52)	30 (15, 61)	28 (14, 50)	33 (18, 69)	24 (13, 52)	33 (18, 69)	24 (13, 52)
EF after CRT (%)	40 (9)	31 (9)	40 (11)	34 (9)	41 (10)	34 (10)	38 (11)	32 (7)	41 (10)	34 (10)	41 (10)	34 (10)
LVESV after CRT (ml)	103 (77, 124)	110 (96, 173)	94 (74, 125)	110 (91, 140)	99 (74, 135)	108 (91, 140)	105 (78, 135)	110 (85, 140)	94 (74, 125)	110 (91, 140)	94 (74, 135)	110 (91, 140)
NYHA after CRT												
I (%)	46/68 (68)	10/41 (24)	31/42 (74)	25/67 (37)	31/43 (72)	25/66 (38)	51/85 (60)	5/24 (21)	30/42 (71)	26/67 (39)	31/42 (74)	25/67 (37)
II (%)	11/68 (16)	6/41 (15)	5/42 (12)	12/67 (18)	5/43 (12)	12/66 (18)	10/85 (12)	7/24 (29)	5/42 (12)	12/67 (18)	5/42 (12)	12/67 (18)
III (%)	8/68 (12)	22/41 (54)	3/42 (7.1)	27/67 (40)	4/43 (9.3)	26/66 (39)	18/85 (21)	12/24 (50)	4/42 (9.5)	26/67 (39)	3/42 (7.1)	27/67 (40)
IV (%)	3/68 (4.4)	3/41 (7.3)	3/42 (7.1)	3/67 (4.5)	3/43 (7.0)	3/66 (4.5)	6/85 (7.1)	0/24 (0)	3/42 (7.1)	3/67 (4.5)	3/42 (7.1)	3/67 (4.5)
Response to CRT (%)	52/68 (76)	12/41 (29)	30/42 (71)	34/67 (51)	32/43 (74)	32/66 (48)	54/85 (64)	10/24 (42)	31/42 (74)	33/67 (49)	32/42 (76)	32/67 (48)
Death (%)	5/68 (7.4)	10/41 (24)	4/42 (9.5)	11/67 (16)	4/43 (9.3)	11/66 (17)	10/85 (12)	5/24 (21)	4/42 (9.5)	11/67 (16)	4/42 (9.5)	11/67 (16)
Hospitalizations												
0 (%)	54/68 (79)	16/41 (39)	33/42 (79)	37/67 (55)	34/43 (79)	36/66 (55)	56/85 (66)	14/24 (58)	33/42 (79)	37/67 (55)	34/42 (81)	36/67 (54)
1 (%)	11/68 (16)	13/41 (32)	6/42 (14)	18/67 (27)	6/43 (14)	18/66 (27)	18/85 (21)	6/24 (25)	5/42 (12)	19/67 (28)	5/42 (12)	19/67 (28)
2 (%)	1/68 (1.5)	4/41 (9.8)	0/42 (0)	5/67 (7.5)	0/43 (0)	5/66 (7.6)	2/85 (2.4)	3/24 (13)	1/42 (2.4)	4/67 (6.0)	0/42 (0)	5/67 (7.5)
3 (%)	1/68 (1.5)	4/41 (9.8)	2/42 (4.8)	3/67 (4.5)	2/43 (4.7)	3/66 (4.5)	5/85 (5.9)	0/24 (0)	2/42 (4.8)	3/67 (4.5)	2/42 (4.8)	3/67 (4.5)
4 (%)	1/68 (1.5)	1/41 (2.4)	1/42 (2.4)	1/67 (1.5)	1/43 (2.3)	1/66 (1.5)	2/85 (2.4)	0/24 (0)	1/42 (2.4)	1/67 (1.5)	1/42 (2.4)	1/67 (1.5)
5 (%)	0/68 (0)	1/41 (2.4)	0/42 (0)	1/67 (1.5)	0/43 (0)	1/66 (1.5)	1/85 (1.2)	0/24 (0)	0/42 (0)	1/67 (1.5)	0/42 (0)	1/67 (1.5)
7 (%)	0/68 (0)	1/41 (2.4)	0/42 (0)	1/67 (1.5)	0/43 (0)	1/66 (1.5)	0/85 (0)	1/24 (4.2)	0/42 (0)	1/67 (1.5)	0/42 (0)	1/67 (1.5)
8 (%)	0/68 (0)	1/41 (2.4)	0/42 (0)	1/67 (1.5)	0/43 (0)	1/66 (1.5)	1/85 (1.2)	0/24 (0)	0/42 (0)	1/67 (1.5)	0/42 (0)	1/67 (1.5)

Abbreviations: AF, atrial fibrillation; AHA, American Heart Association; ARNI, angiotensin receptor neprilysin inhibitor; BMI, body mass index; CCB, calcium channel blocker; CRT, cardiac resynchronization therapy; EF, ejection fraction; ESC, European Society of Cardiology; HF, heart failure; LA, left atrium; LAVI, left atrial volume index; LBBB, left bundle branch block; LVEDD, left ventricular end‐diastolic diameter; LVEDV, left ventricular end‐diastolic volume; LVEF, left ventricular ejection fraction; LVESD, left ventricular end‐systolic diameter; LVESV, left ventricular end‐systolic volume; MRA, mineralocorticoid receptor antagonist; NYHA, New York Heart Association; PR, PR interval; QRS, QRS duration; QTc, corrected QT interval; RAAS, renin−angiotensin−aldosterone system; RWPT, R‐wave peak time; SGLT2i, sodium‐glucose cotransporter type 2 inhibitor; SR, sinus rhythm; WHO, World Health Organization.

Regarding ECG classification, 68 patients (62%) fulfilled Strauss criteria, 42 (39%) Marriott criteria, 43 (39%) Perrin criteria, 85 (78%) ESC 2013 criteria, 42 (39%) ESC 2021 criteria, and 42 (39%) WHO/AHA criteria. The distribution of LBBB criteria across the strata of Strauss LBBB (Strauss vs. non‐Strauss) is presented in Supporting Information S1: Table S2. Accordingly, a detailed table with the pairwise agreement coefficients between the examined LBBB criteria is reported in Supporting Information S1: Table S3. Of the examined criteria, the maximum agreement between Strauss LBBB and any other criterion was moderate, observed for LBBB ESC 2013 (Gwet's AC1, 0.51; 95% CI, 0.34–0.68). The median follow‐up duration was 30 months (IQR 14–60), with a maximum follow‐up of 125 months, as reflected in the Kaplan–Meier analyses. During follow‐up, 15 deaths and 39 HF hospitalizations were recorded.

CRT response rates were 52/68 (76%) for Strauss‐LBBB, 30/42 (71%) for Marriott‐LBBB, 32/43 (74%) for Perrin‐LBBB, 54/85 (64%) for ESC 2013‐LBBB, 31/42 (74%) for ESC 2021‐LBBB, and 32/42 (76%) for WHO/AHA‐LBBB. Mean LVEF after implantation increased to 43%. Most responders improved to NYHA class I (53/64, 83%). Responders experienced fewer deaths (2/64 [3.1%] vs. 13/45 [29%]; *p* < 0.001) and fewer HF hospitalizations (7/64 [11%] vs. 32/45 [71%]; *p* < 0.001) compared with nonresponders. Regarding electrical resynchronization parameters, Strauss LBBB demonstrated both a large QRS narrowing in patients fulfilling the Strauss definition coupled with a relatively small QRS change in those with non‐Strauss LBBB [−41 ± 17 vs. −23 ± 17 ms; *p* < 0.001], with a similar pattern in the relative QRS index (%) (−24 [9] vs. −15 [10], *p* < 0.001). For the other definitions, either QRS narrowing among criterion‐positive patients was less pronounced or QRS narrowing among criterion‐negative patients remained substantial; neither feature was observed simultaneously (Supporting Information S1: Table S4).

### 3.2 LBBB Definitions and CRT Response

**Figure 2:**
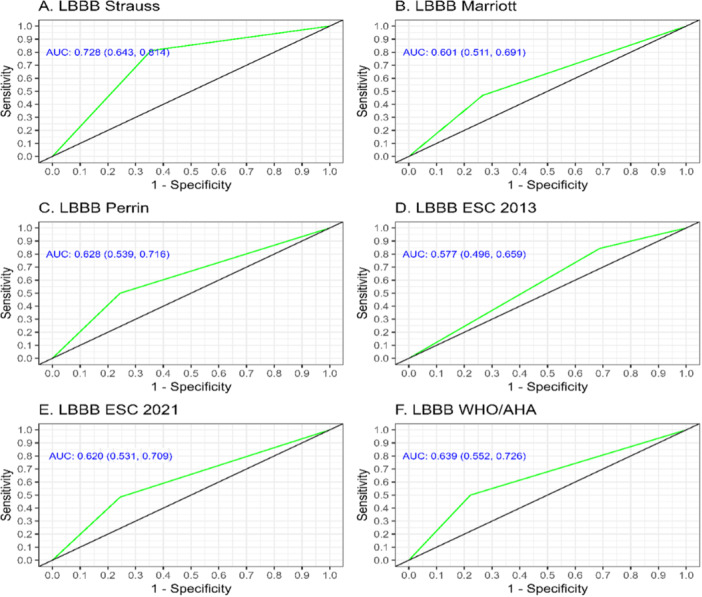
Receiver operating characteristic curves for prediction of CRT response according to LBBB definitions. The Strauss definition showed the highest diagnostic performance (AUC 0.728, 95% CI 0.643–0.814). (A) LBBB Strauss: ROC curve for the Strauss LBBB definition. This criterion showed the best discriminatory performance, with an AUC of 0.728. (B) LBBB Marriott: ROC curve for the Marriott LBBB definition. This criterion showed modest discriminatory performance, with an AUC of 0.601. (C) LBBB Perrin: ROC curve for the Perrin LBBB definition. This criterion showed moderate discriminatory performance, with an AUC of 0.628. (D) LBBB ESC 2013: ROC curve for the 2013 ESC LBBB definition. This criterion showed limited discriminatory performance, with an AUC of 0.577. (E) LBBB ESC 2021: ROC curve for the 2021 ESC LBBB definition. This criterion showed modest discriminatory performance, with an AUC of 0.620. (F) LBBB WHO/AHA: ROC curve for the WHO/AHA LBBB definition. This criterion showed moderate discriminatory performance, with an AUC of 0.639. AHA, American Heart Association; AUC, area under the curve; CI, confidence interval; ESC, European Society of Cardiology; WHO, World Health Organization.

Unadjusted diagnostic accuracy metrics are shown in Table 4, with ROC curves illustrated in Figure 2. Strauss‐LBBB showed the highest AUC (0.728, 95% CI 0.643–0.814). The respective AUC values were 0.639 (95% CI 0.552–0.726) for WHO/AHA, 0.628 (95% CI 0.539–0.716) for Perrin, 0.620 (95% CI 0.531–0.709) for ESC 2021, 0.601 (95% CI 0.511–0.691) for Marriott, and 0.577 (95% CI 0.496–0.659) for ESC 2013. Pairwise comparisons are presented in Supporting Information S1: Table S5. Strauss‐LBBB had a significantly higher AUC than the Marriott (difference 0.127, *p* = 0.007), Perrin (0.101, *p* = 0.026), ESC 2013 (0.151, *p* = 0.003), and ESC 2021 (0.109, *p* = 0.024) definitions, whereas the difference versus WHO/AHA was borderline (0.090, *p* = 0.053).

**Table 4:** Unadjusted diagnostic accuracy measures for predicting CRT response by different LBBB criteria.

LBBB definition	Sensitivity	Specificity	PPV	NPV	AUC (95% CI)	*p* value
Strauss	0.81	0.64	0.76	0.71	0.728 (0.643, 0.814)	< 0.001
Marriott	0.47	0.73	0.71	0.49	0.601 (0.511, 0.691)	0.027
Perrin	0.50	0.76	0.74	0.52	0.628 (0.539, 0.716)	0.005
ESC 2013	0.84	0.31	0.64	0.58	0.577 (0.496, 0.659)	0.063
ESC 2021	0.48	0.76	0.74	0.51	0.620 (0.531, 0.709)	0.008
WHO/AHA	0.50	0.78	0.76	0.52	0.639 (0.552, 0.726)	0.002

Abbreviations: AHA, American Heart Association; AUC, area under the curve; CI, confidence interval; ESC, European Society of Cardiology; LBBB, left bundle branch block; NPV, negative predictive value; PPV, positive predictive value; WHO, World Health Organization.

**Figure 3:**
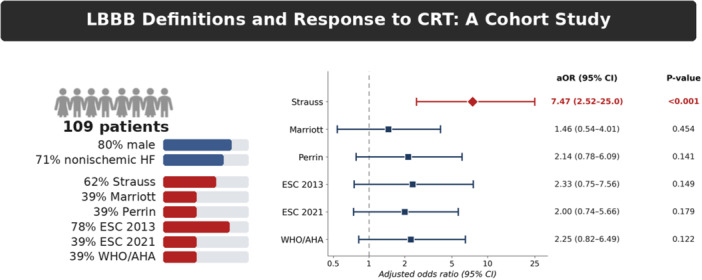
Study cohort overview and multivariable logistic regression analysis of LBBB definitions and CRT response. The left panel summarizes the baseline characteristics of the study population (*n* = 109) and the proportion of patients fulfilling each LBBB definition. The right panel presents the multivariable logistic regression analysis; only the Strauss definition was independently associated with response (aOR 7.47, *p* < 0.001). Each model was adjusted for age, sex, heart failure type, paroxysmal atrial fibrillation, New York Heart Association class, left ventricular ejection fraction, left ventricular end‐systolic volume, baseline QRS duration, and use of angiotensin receptor‐neprilysin inhibitors. aOR, adjusted odds ratio; CI, confidence interval; CRT, cardiac resynchronization therapy; LBBB, left bundle branch block.

In multivariable logistic regression models, the Strauss definition was significantly associated with CRT response (OR 7.47, 95% CI 2.52–25.0; *p* < 0.001), whereas associations for the remaining definitions did not reach statistical significance (Figure 3, Supporting Information S1: Table S6). In sensitivity analysis for Strauss LBBB, an unmeasured confounder would need to be associated with both Strauss and CRT response by an OR of at least 2.55 to render the association nonsignificant, and 4.91 to fully explain it.

### 3.3 LBBB Definitions and Mortality

**Figure 4:**
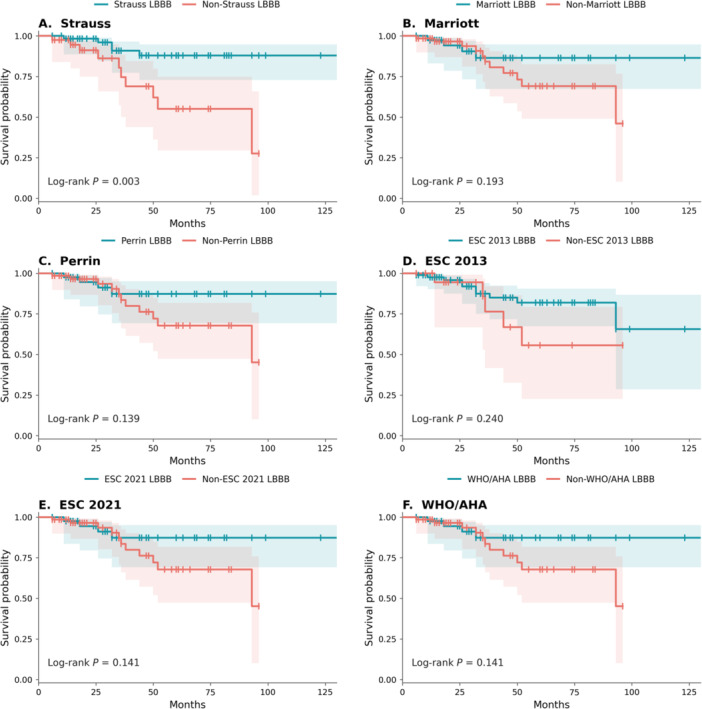
Kaplan–Meier survival curves according to LBBB definitions. Strauss‐defined LBBB was associated with a higher observed survival probability (log‐rank *p* = 0.003). (A) Strauss LBBB versus non‐Strauss LBBB, (B) Marriott LBBB versus non‐Marriott LBBB, (C) Perrin LBBB versus non‐Perrin LBBB, (D) ESC 2013 LBBB versus non‐ESC 2013 LBBB, (E) ESC 2021 LBBB versus non‐ESC 2021 LBBB, (F) WHO/AHA LBBB versus non‐WHO/AHA LBBB. Number of events: 15 deaths during follow‐up. AHA, American Heart Association; ESC, European Society of Cardiology; WHO, World Health Organization.

Kaplan–Meier survival curves according to LBBB definitions are presented in Figure 4. Among the examined definitions, Strauss‐LBBB was the only definition significantly associated with mortality in this cohort (log‐rank *p* = 0.003).

**Figure 5:**
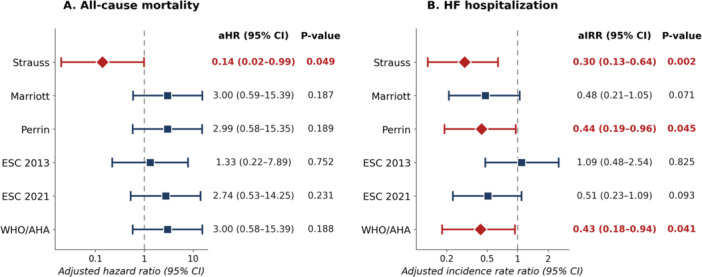
Association between LBBB definitions and clinical outcomes. (A) All‐cause mortality was assessed by Cox regression. (B) Heart failure hospitalization rates assessed by negative binomial regression. AHA, American Heart Association; aHR, adjusted hazard ratio; aIRR, adjusted incidence rate ratio; CI, confidence interval; ESC, European Society of Cardiology; HR, hazard ratio; IRR, incidence rate ratio; WHO, World Health Organization.

In adjusted Cox regression analyses (Figure 5A, Supporting Information S1: Table S7), Strauss‐LBBB was associated with a lower observed mortality hazard (HR 0.14, 95% CI 0.02–0.99; *p* = 0.049), whereas no significant associations were observed for Marriott, Perrin, ESC 2013, ESC 2021, or WHO/AHA definitions. In sensitivity analysis, an unmeasured confounder associated with both Strauss and mortality by an HR of at least 1.11 could move the CI to include the null, whereas a confounder associated with both by an HR of 13.77 would be required to fully explain away the observed association.

### 3.4 LBBB Definitions and HF Hospitalizations

Strauss‐LBBB demonstrated the greatest association with hospitalization (IRR 0.30, 95% CI 0.13–0.64; *p* = 0.002). Among the other LBBB criteria, only Perrin (IRR = 0.44, 95% CI 0.19–0.96; *p* = 0.045) and WHO/AHA (IRR = 0.43, 95% CI 0.18–0.94; *p* = 0.041) demonstrated a significant association with fewer HF hospitalizations. Nonsignificant associations were noted for Marriott, ESC 2013, and ESC 2021 LBBB (Figure 5B, Supporting Information S1: Table S8). An unmeasured confounder associated with both Strauss LBBB and HF hospitalization by an IRR of at least 2.50 could move the CI toward the null, whereas a confounder associated with both by an IRR of 6.12 would be required to fully explain away the observed association.

## 4 Discussion

In this single‐center observational cohort of patients with HF undergoing CRT‐D implantation, we evaluated the association between six electrocardiographic definitions of LBBB and clinical outcomes and found that stricter LBBB criteria were associated with a higher likelihood of reverse remodeling. The main findings of this study can be summarized as follows:

i. Strauss‐defined LBBB was independently associated with echocardiographic response to CRT in this cohort.

ii. The presence of Strauss‐defined LBBB was associated with lower HF hospitalization rates and showed a favorable association with survival outcomes in this cohort.

However, given the observational design and relatively small sample size, these findings should be interpreted cautiously. In the context of existing evidence, several studies have examined the impact of different LBBB definitions on CRT response, with heterogeneous findings. In a large multicenter cohort of 1492 patients, van Stipdonk et al. showed that stricter LBBB criteria were associated with improved prediction of CRT response compared with conventional definitions [26]. Similarly, Kashtanova et al. reported that strict morphological criteria identified patients with delayed transseptal conduction and were associated with superior echocardiographic response [27].

García‐Seara et al. reported that patients with Strauss‐LBBB and Perrin‐LBBB had greater echocardiographic response and lower rates of HF hospitalizations compared to patients with non‐true LBBB [28]. However, CRT response in that study was defined as an increase in LVEF ≥ 5% or a reduction in LVESV ≥ 15%, which differs from the response definition used in the present study. In a similar cohort of 335 patients, the Strauss and AHA/WHO definitions showed no difference in CRT response, defined as ≥ 15% reduction in LVESV [10]. Similarly, Caputo and colleagues evaluated multiple LBBB definitions (AHA/WHO, ESC 2006, ESC 2009, ESC 2013, and Strauss) and reported statistical significance only for ESC 2009 and ESC 2013 criteria; however, the implantation rate of quadripolar LV leads was < 5%, which may limit comparisons with contemporary cohorts [8].

Conversely, Mascioli et al. reported improved echocardiographic response and survival among patients fulfilling Strauss criteria, while Tian et al. reported that Strauss‐LBBB morphology may identify patients with super‐response to CRT [11, 12]. Additionally, a recent retrospective analysis showed greater echocardiographic response when strict LBBB criteria were applied [29]. Mugnai et al. further reported that defining LBBB according to Strauss criteria yielded an OR of 4.40 for CRT response compared with 1.93 for guideline‐based LBBB definitions [30]. These findings are broadly consistent with the results of the present study, in which the Strauss criteria were independently associated with CRT response (OR 7.47). From a pathophysiological perspective, Strauss‐LBBB may represent a more specific electrocardiographic manifestation of mechanical desynchrony, potentially explaining the higher likelihood of reverse remodeling [31, 32]. Nevertheless, variations in patient selection, device technology, response definitions, and ECG interpretation may account for the differences observed across studies.

The superior performance of the Strauss definition may be explained by its underlying electrophysiological rationale. By combining longer, sex‐specific QRS‐duration thresholds (≥ 130 ms in women and ≥ 140 ms in men) with the mandatory presence of mid‐QRS notching or slurring in at least two leads, the Strauss criteria are designed to identify complete interruption of left‐sided conduction with transseptal activation delay, rather than left ventricular hypertrophy or non‐specific intraventricular conduction delay that may mimic an LBBB pattern [14, 31]. This pattern reflects a phenotype more consistent with advanced left‐sided conduction delay with a greater degree of baseline electrical dyssynchrony, the substrate most amenable to correction by biventricular pacing. In our cohort, Strauss‐defined LBBB was the only criterion that combined marked QRS narrowing in patients who met it with minimal narrowing in those who did not (Supporting Information S1: Table S4), in line with the premise that stricter morphological criteria select patients whose dyssynchrony is more correctable and who therefore achieve greater reverse remodeling and more favorable observed clinical outcomes. The agreement analysis pointed in the same direction (Supporting Information S1: Table S3): Strauss LBBB agreed only weakly to moderately with every other definition (Gwet's AC1 0.38–0.51), whereas the other criteria, with the exception of ESC 2013, agreed closely among themselves (0.91–0.98). The Strauss definition, therefore, appears to identify a distinct electrocardiographic phenotype rather than a stricter variant of the same entity.

### 4.1 Hospitalizations and Mortality

#### 4.1.1 Hospitalizations

Among the examined definitions, Strauss, Perrin, and WHO/AHA criteria were associated with lower HF hospitalization rates, with the strongest association observed for Strauss‐defined LBBB. This finding may be partly explained by the higher proportion of responders within the Strauss‐LBBB subgroup, suggesting that stricter electrocardiographic criteria may better identify patients who derive sustained clinical benefit after CRT implantation. Previous studies have similarly reported lower hospitalization rates for HF decompensation among patients fulfilling Strauss criteria compared with non‐Strauss LBBB patterns [8, 33]. However, given the observational design and limited number of clinical events, these findings should be interpreted cautiously and considered hypothesis‐generating.

#### 4.1.2 Mortality

Regarding survival outcomes, prior investigations have reported mixed findings. Caputo et al. observed a survival benefit associated with the ESC 2013 and Strauss definitions [8], whereas Jastrzebski et al. demonstrated improved mortality prediction using Strauss criteria compared with conventional definitions [9]. In the present cohort, only Strauss‐LBBB showed a statistically significant association with survival; however, the relatively small number of deaths limits definitive conclusions. The favorable survival signal observed in our analysis may be related to improved reverse remodeling and reduced HF progression among responders, as previously suggested by larger multicenter studies [22, 34].

### 4.2 Implications for Clinical Practice and Future Research

These findings should be interpreted within the broader context of HF management, where accurate identification of patients most likely to benefit from device therapy remains a clinical priority. Current international guidelines recommend CRT primarily on the basis of QRS duration and LBBB morphology; however, the optimal electrocardiographic definition of LBBB remains debated. In current clinical practice, CRT constitutes an important therapeutic option for selected patients with HF and reduced ejection fraction [1]. In successfully treated patients, improvements may extend beyond symptoms and quality of life to include favorable effects on clinical outcomes [35, 36]. Nevertheless, this therapeutic intervention is also associated with certain periprocedural [37] and long‐term complications as well as significant costs [38]. Considerable heterogeneity in electrocardiographic definitions and response criteria continues to complicate the field [39]. The present findings should therefore be interpreted within the context of a single‐center observational cohort.

In line with previous observational evidence, our findings suggest that stricter morphological definitions of LBBB, particularly the Strauss criteria, may better identify patients with a higher probability of CRT response. In contrast, the ESC 2021 definition was not independently associated with response or clinical outcomes in this cohort [18]. Recent data suggest that highly restrictive definitions may reduce the number of patients classified as LBBB, potentially excluding individuals with clinically relevant mechanical desynchrony [40]. The superior discriminative performance and stronger association observed for Strauss‐LBBB in our analysis support the concept that refined ECG characterization may improve patient selection; however, these observations should not be interpreted as definitive evidence to modify current guideline recommendations.

Differences between studies may be explained by variations in ischemic versus non‐ischemic cardiomyopathy, response definitions, and interobserver variability in ECG interpretation. Indeed, prior work has shown that a considerable proportion of ECGs may be interpreted differently when applying distinct LBBB criteria [41]. Emerging computational ECG approaches, including artificial intelligence‐based models such as FactorECG, may further refine the prediction of CRT response in future investigations [42]. Therefore, the present findings should be viewed as hypothesis‐generating and may help inform future prospective studies evaluating electrocardiographic markers of CRT response.

### 4.3 Strengths and Limitations

This study provides a comprehensive evaluation of multiple LBBB definitions in relation to both echocardiographic and clinical outcomes in a contemporary CRT cohort. The simultaneous assessment of response, hospitalization, and survival, as well as the use of both unadjusted and adjusted analyses, represent important strengths.

Several limitations should be acknowledged. The relatively small sample size, single‐center design, and limited number of clinical events limit the strength of our inferences and the generalizability of our findings to other cohorts. In addition, the small sample size precluded more extensive sensitivity analyses. For this reason, the analysis of mortality and hospitalization should be considered exploratory. Competing risk analysis for hospitalization was not feasible due to unavailable time‐to‐event data. Interobserver variability in ECG interpretation cannot be fully excluded, and echocardiographic assessments were not performed in a blinded fashion. As this analysis was restricted to an LBBB‐selected cohort, the findings may not be generalizable to patients with non‐LBBB conduction abnormalities, who typically demonstrate lower response rates and worse outcomes after CRT.

Detailed procedural information, including left ventricular lead polarity (bipolar vs. quadripolar) and device programming or optimization strategies, was not systematically recorded throughout the enrollment period and could not be accounted for; nonetheless, the left ventricular lead was positioned in a lateral or posterolateral vein in the large majority of patients, without significant differences across response groups (Table 2). Moreover, patients were enrolled over a 10‐year period (2013–2023), during which guideline‐directed medical therapy evolved substantially, most notably with the introduction and broader uptake of angiotensin receptor–neprilysin inhibitors and sodium–glucose cotransporter‐2 inhibitors, together with refinements in CRT implantation technique and device programming, including the increasing use of quadripolar leads. Although the use of contemporary medical therapy and left ventricular lead position were comparable across LBBB strata, residual temporal confounding cannot be excluded and should be considered when interpreting our findings.

## 5 Conclusions

Among the evaluated electrocardiographic definitions, Strauss‐defined LBBB was independently associated with echocardiographic response and more favorable observed clinical outcomes in this single‐center cohort. These findings suggest that stricter morphological criteria may help refine patient selection for CRT; however, confirmation in larger prospective studies is required before clinical implementation.

## Author Contributions

Athanasios Saplaouras conceived and designed the study, contributed to data collection, and drafted the manuscript. Konstantinos Pamporis performed the statistical analyses and contributed to data interpretation. Panagiotis Mililis, Stavroula Koskina, Athanasios Makris, and Sokratis Oikonomou contributed to patient recruitment and data acquisition. Theodoros Efremidis, Athena Batsoul, and Ourania Kariki contributed to data interpretation and critical revision of the manuscript. George Bazoukis, Sotirios Xydonas, and Stylianos Dragasis performed electrocardiographic analyses. Theodoros Karamitsos, Christodoulos Papadopoulos, and Nikolaos Fragakis contributed to study supervision and critical revision for important intellectual content. Michael Efremidis and Konstantinos P. Letsas contributed to study design, overall supervision, and final approval of the manuscript. All authors read and approved the final version of the manuscript.

## Funding

The authors have nothing to report.

## Conflicts of Interest

The authors declare no conflicts of interest.

## Supporting information

Supporting File

## Data Availability

The data underlying this article will be shared on reasonable request to the corresponding author.
