# Supplementary material for: Strauss‐Defined LBBB Identifies Patients With Improved Outcomes After CRT‐D: A Comparative Cohort Study
Source: Clin Cardiol. 2026 Jul 6;49(7):e70408. doi: 10.1002/clc.70408 (PMC13335088; doi:10.1002/clc.70408)
Supplement: Supplementary file 1 — Supporting File [file CLC-49-e70408-s001.docx]

**Supplementary Material**

**Table S.1 :** Supplemental for statistical analysis

| **LBBB and response to CRT (logistic regression):** |
| --- |
| In the multivariable logistic regression for CRT response, possible confounders were combined with variables identified through univariate modeling (p<0.20) [1-3]. The final models were constructed using a non-automated backward elimination technique and comparison of nested models with the likelihood ratio test. All previously identified possible confounders were retained independently of their level of significance. All regression models were finally adjusted for sex, age, LVEF, LVESV, HF type, NYHA class, paroxysmal AF, QRS duration and use of ARNI. All models were thoroughly assessed for the validity of logistic regression assumptions. |
| **LBBB morphologies and all-cause death (Cox regression)** |
| For the multivariable Cox regression model, previously reported predictors were identified in the literature and a final model was created by combining these predictors with the results from univariate modeling (p<0.20).[4] Afterwards, a non-automated backward elimination technique via the comparison of nested models with the likelihood ratio test was implemented. All previously identified possible confounders were retained independently of their level of significance in the current dataset. The final Cox models were adjusted for age, sex, HF type, paroxysmal AF, NYHA class, LVEF, QRS duration, LVESV and use of b-blockers. Proportionality was assessed for each variable separately using log-minus-log plots along with individual and global goodness-of-fit test based on Schoenfeld residuals. Potentially influential values were assessed using normalized transformations of the Martingale residuals. |
| **LBBB morphologies and hospitalizations (negative binomial regression)** |
| For the analysis of hospitalizations, multivariable negative binomial regression models were used to account for the identified overdispersion. Previously identified predictors were again combined with variables from univariate modeling (p<0.20). A non-automated backward elimination technique via the comparison of nested models with the likelihood ratio test was used. All previously identified possible confounders were retained independently of their level of significance. Negative binomial models were adjusted for age, sex, HF type, paroxysmal AF, NYHA class, LVEF, QRS duration, LVESV, MRA and antiarrhythmics. No zero inflation was identified. |

Abbreviations: LVEF, left ventricular ejection fraction; LVESV, left ventricular end-systolic volume; HF, heart failure; NYHA, New York Heart Association; AF, atrial fibrillation; ARNI, angiotensin receptor neprilysin inhibitors; MRA, mineralocorticoid receptor antagonists.

**Table S.2** Distribution of LBBB criteria in strata based on Strauss definition

| **LBBB criterion** | **Strauss**  **N = 68** | **Non-Strauss**  **N = 41** | **p-value** |
| --- | --- | --- | --- |
| LBBB Marriott | 38/68 (56%) | 4/41 (9.8%) | <0.001 |
| LBBB Perrin | 40/68 (59%) | 3/41 (7.3%) | <0.001 |
| LBBB ESC 2013 | 61/68 (90%) | 24/41 (59%) | <0.001 |
| LBBB ESC 2021 | 38/68 (56%) | 4/41 (9.8%) | <0.001 |
| LBBB WHO/AHA | 39/68 (57%) | 3/41 (7.3%) | <0.001 |

Abbreviations: LBBB, left bundle branch block; ESC, European Society of Cardiology; WHO, World Health Organization; AHA, American Heart Association.

**Table S.3.** Gwet’s AC1 between different LBBB criteria

| **LBBB criteria** | LBBB Strauss | LBBB Marriott | LBBB Perrin | LBBB ESC 2013 | LBBB ESC 2021 | LBBB WHO/AHA |
| --- | --- | --- | --- | --- | --- | --- |
| LBBB Strauss |  | 0.38 (0.199,0.553) | 0.43 (0.259,0.603) | 0.51 (0.34,0.681) | 0.38 (0.199,0.553) | 0.41 (0.239,0.587) |
| LBBB Marriott | 0.38 (0.199,0.553) |  | 0.91 (0.836,0.989) | 0.23 (0.041,0.423) | 0.93 (0.862,0.999) | 0.93 (0.862,0.999) |
| LBBB Perrin | 0.43 (0.259,0.603) | 0.91 (0.836,0.989) |  | 0.22 (0.024,0.409) | 0.95 (0.888,1) | 0.98 (0.948,1) |
| LBBB ESC 2013 | 0.51 (0.34,0.681) | 0.23 (0.041,0.423) | 0.22 (0.024,0.409) |  | 0.23 (0.041,0.423) | 0.23 (0.041,0.423) |
| LBBB ESC 2021 | 0.38 (0.199,0.553) | 0.93 (0.862,0.999) | 0.95 (0.888,1) | 0.23 (0.041,0.423) |  | 0.97 (0.916,1) |
| LBBB WHO/AHA | 0.41 (0.239,0.587) | 0.93 (0.862,0.999) | 0.98 (0.948,1) | 0.23 (0.041,0.423) | 0.97 (0.916,1) |  |

Abbreviations: LBBB, left bundle branch block; ESC, European Society of Cardiology; WHO, World Health Organization; AHA, American Heart Association.

**Table S.4.** Differences in electrical resynchronization parameters across LBBB criteria

| **Variable** | LBBB Strauss | | | LBBB Marriott | | | LBBB Perrin | | | LBBB ESC 2013 | | | LBBB ESC 2021 | | | LBBB WHO/AHA | | |
| --- | --- | --- | --- | --- | --- | --- | --- | --- | --- | --- | --- | --- | --- | --- | --- | --- | --- | --- |
|  | **Yes (n=68)** | **No (n=41)** | **p** | **Yes (n=42)** | **No (n=67)** | **p** | **Yes (n=43)** | **No (n=66)** | **p** | **Yes (n=85)** | **No (n=24)** | **p** | **Yes (n=42)** | **No (n=67)** | **p** | **Yes (N=42)** | **No (n=67)** | **p** |
| QRS difference (ms) | -41 (17) | -23 (17) | <0.001 | -42 (15) | -30 (20) | <0.001 | -42 (16) | -30 (19) | <0.001 | -36 (19) | -28 (18) | 0.056 | -42 (15) | -30 (20) | <0.001 | -43 (15) | -30 (19) | <0.001 |
| QRS index (%) | -24 (9) | -15 (10) | <0.001 | -24 (8) | -18 (11) | 0.002 | -24 (8) | -18 (11) | 0.002 | -22 (10) | -17 (10) | 0.053 | -25 (8) | -18 (11) | <0.001 | -25 (8) | -18 (11) | <0.001 |

Abbreviations: LBBB, left bundle branch block; ESC, European Society of Cardiology; WHO, World Health Organization; AHA, American Heart Association.

**Table S.5**: Pairwise comparisons of diagnostic accuracy of different left bundle branch block (LBBB) criteria for predicting response after CRT

| **LBBB criteria** | LBBB Strauss | LBBB Marriott | LBBB Perrin | LBBB ESC 2013 | LBBB ESC 2021 | LBBB WHO/AHA |
| --- | --- | --- | --- | --- | --- | --- |
| LBBB Strauss | --- | 0.127 (0.035, 0.220), **p=0.007** | 0.101 (0.012, 0.189), **p=0.026** | 0.151 (0.050, 0.252), **p=0.003** | 0.109 (0.014, 0.203), **p=0.024** | 0.090 (-0.001, 0.180), p=0.053 |
| LBBB Marriott |  | --- | -0.027 (-0.070, 0.017), p=0.230 | 0.024 (-0.071, 0.118), p=0.624 | -0.019 (-0.060, 0.022), p=0.365 | -0.038 (-0.075, -0.001), **p=0.047** |
| LBBB Perrin |  |  | --- | 0.050 (-0.049, 0.149), p=0.319 | 0.008 (-0.027, 0.043), p=0.659 | -0.011 (-0.033, 0.011), p=0.317 |
| LBBB ESC 2013 |  |  |  | --- | -0.043 (-0.137, 0.052), p=0.377 | -0.061 (-0.156, 0.033), p=0.201 |
| LBBB ESC 2021 |  |  |  |  | --- | -0.019 (-0.046, 0.008), p=0.164 |
| LBBB WHO/AHA |  |  |  |  |  | --- |

Results are presented as the difference in the area under the curve between the criterion in each row minus the criterion in each column accompanied by 95% confidence intervals and the p-value of the DeLong test. Abbreviations: LBBB, left bundle branch block; ESC, European Society of Cardiology; WHO, World Health Organization; AHA, American Heart Association.

**Table S.6**: Multivariable logistic regression for response to cardiac resynchronization therapy by different criteria of left bundle branch block (LBBB)

| **Variable** | **LBBB Strauss** | | **LBBB Marriott** | | **LBBB Perrin** | | **LBBB ESC 2013** | | **LBBB ESC 2021** | | **LBBB WHO/AHA** | |
| --- | --- | --- | --- | --- | --- | --- | --- | --- | --- | --- | --- | --- |
|  | **aOR (95% CI)** | **p-value** | **aOR (95% CI)** | **p-value** | **aOR (95% CI)** | **p-value** | **aOR (95% CI)** | **p-value** | **aOR (95% CI)** | **p-value** | **aOR (95% CI)** | **p-value** |
| LBBB | 7.47 (2.52, 25.0) | <0.001 | 1.46 (0.54, 4.01) | 0.454 | 2.14 (0.78, 6.09) | 0.141 | 2.33 (0.75, 7.56) | 0.149 | 2.00 (0.74, 5.66) | 0.179 | 2.25 (0.82, 6.49) | 0.122 |
| Sex (Male vs Female) | 0.23 (0.05, 0.91) | 0.047 | 0.22 (0.05, 0.80) | 0.031 | 0.22 (0.05, 0.84) | 0.035 | 0.24 (0.05, 0.89) | 0.043 | 0.22 (0.05, 0.84) | 0.036 | 0.23 (0.05, 0.86) | 0.039 |
| Age (years) | 1.00 (0.95, 1.05) | >0.9 | 1.01 (0.96, 1.06) | 0.8 | 1.01 (0.96, 1.06) | 0.7 | 1.01 (0.96, 1.06) | 0.7 | 1.01 (0.96, 1.06) | 0.7 | 1.01 (0.96, 1.06) | 0.7 |
| LVEF (%) | 0.98 (0.87, 1.10) | 0.7 | 1.02 (0.92, 1.14) | 0.7 | 1.02 (0.91, 1.13) | 0.7 | 1.01 (0.90, 1.12) | 0.9 | 1.02 (0.91, 1.13) | 0.8 | 1.02 (0.91, 1.13) | 0.8 |
| LVESV (ml) | 1.00 (0.99, 1.01) | 0.9 | 1.00 (0.99, 1.01) | >0.9 | 1.00 (0.99, 1.01) | >0.9 | 1.00 (0.99, 1.01) | >0.9 | 1.00 (0.99, 1.01) | >0.9 | 1.00 (0.99, 1.01) | >0.9 |
| HF type (Non-ischemic vs Ischemic) | 3.98 (1.39, 12.3) | 0.012 | 4.53 (1.69, 13.1) | 0.004 | 4.65 (1.72, 13.6) | 0.003 | 4.43 (1.65, 12.8) | 0.004 | 4.70 (1.74, 13.8) | 0.003 | 4.55 (1.68, 13.4) | 0.004 |
| NYHA (III/IV vs II) | 1.17 (0.29, 4.64) | 0.8 | 1.20 (0.32, 4.40) | 0.8 | 1.33 (0.35, 5.02) | 0.7 | 1.38 (0.36, 5.22) | 0.6 | 1.31 (0.35, 4.85) | 0.7 | 1.28 (0.33, 4.88) | 0.7 |
| Paroxysmal AF | 0.34 (0.12, 0.95) | 0.044 | 0.39 (0.15, 0.99) | 0.051 | 0.38 (0.14, 0.98) | 0.049 | 0.34 (0.12, 0.87) | 0.028 | 0.38 (0.14, 0.98) | 0.048 | 0.38 (0.14, 0.97) | 0.047 |
| Baseline QRS (ms) | 1.01 (0.98, 1.04) | 0.5 | 1.03 (1.00, 1.06) | 0.037 | 1.03 (1.00, 1.06) | 0.060 | 1.03 (1.01, 1.06) | 0.019 | 1.03 (1.00, 1.06) | 0.050 | 1.03 (1.00, 1.06) | 0.070 |
| ARNI | 0.28 (0.09, 0.81) | 0.023 | 0.29 (0.10, 0.77) | 0.016 | 0.30 (0.10, 0.81) | 0.021 | 0.30 (0.10, 0.82) | 0.022 | 0.30 (0.10, 0.82) | 0.023 | 0.31 (0.11, 0.85) | 0.026 |

Abbreviations: LBBB, left bundle branch block; OR, odds ratio; aOR, adjusted odds ratio; EF, ejection fraction; LVESV, left-ventricular end-systolic volume; HF, heart failure; AF, atrial fibrillation; ECG, electrocardiogram; ARNI, angiotensin receptor neprilysin inhibitor; ESC, European Society of Cardiology; WHO, World Health Organization; AHA, American Heart Association.

**Table S.7:** Results of multivariable Cox regression for survival after CRT by different criteria of left bundle branch block (LBBB)

| **Variable** | **LBBB Strauss** | | **LBBB Marriott** | | **LBBB Perrin** | | **LBBB ESC 2013** | | **LBBB ESC 2021** | | **LBBB WHO/AHA** | |
| --- | --- | --- | --- | --- | --- | --- | --- | --- | --- | --- | --- | --- |
|  | **aHR (95% CI)** | **p-value** | **aHR (95% CI)** | **p-value** | **aHR (95% CI)** | **p-value** | **aHR (95% CI)** | **p-value** | **aHR (95% CI)** | **p-value** | **aHR (95% CI)** | **p-value** |
| LBBB | 0.14 (0.02, 0.99) | 0.049 | 3.00 (0.59, 15.4) | 0.2 | 2.99 (0.58, 15.4) | 0.2 | 1.33 (0.22, 7.89) | 0.8 | 2.74 (0.53, 14.2) | 0.2 | 3.00 (0.58, 15.4) | 0.2 |
| Age (years) | 0.98 (0.92, 1.04) | 0.5 | 0.99 (0.93, 1.06) | 0.8 | 0.99 (0.93, 1.06) | 0.8 | 0.98 (0.92, 1.04) | 0.5 | 0.99 (0.93, 1.06) | 0.8 | 0.99 (0.93, 1.06) | 0.8 |
| Sex (Male vs Female) | 3.05 (0.38, 24.2) | 0.3 | 1.52 (0.30, 7.59) | 0.6 | 1.52 (0.30, 7.57) | 0.6 | 1.39 (0.28, 6.97) | 0.7 | 1.47 (0.30, 7.18) | 0.6 | 1.52 (0.30, 7.59) | 0.6 |
| HF type (Non-ischemic vs Ischemic) | 0.03 (0.00, 0.25) | 0.001 | 0.05 (0.01, 0.21) | <0.001 | 0.05 (0.01, 0.23) | <0.001 | 0.05 (0.01, 0.24) | <0.001 | 0.05 (0.01, 0.23) | <0.001 | 0.05 (0.01, 0.23) | <0.001 |
| Paroxysmal AF | 7.85 (1.12, 55) | 0.040 | 4.24 (0.87, 20.6) | 0.074 | 4.26 (0.87, 20.7) | 0.073 | 3.80 (0.71, 20.5) | 0.12 | 4.44 (0.91, 21.7) | 0.066 | 4.25 (0.87, 20.7) | 0.073 |
| NYHA (III/IV vs II) | 3.54 (0.32, 39) | 0.3 | 4.75 (0.36, 62.5) | 0.2 | 4.82 (0.37, 62.8) | 0.2 | 5.32 (0.32, 89.7) | 0.2 | 5.75 (0.45, 73.2) | 0.2 | 4.78 (0.36, 62.6) | 0.2 |
| LVEF (%) | 0.92 (0.81, 1.04) | 0.2 | 0.84 (0.73, 0.97) | 0.018 | 0.84 (0.73, 0.97) | 0.018 | 0.86 (0.74, 1.01) | 0.062 | 0.84 (0.73, 0.97) | 0.020 | 0.84 (0.73, 0.97) | 0.018 |
| LVESV (ml) | 0.98 (0.97, 1.00) | 0.032 | 0.99 (0.98, 1.00) | 0.080 | 0.99 (0.98, 1.00) | 0.080 | 0.99 (0.98, 1.00) | 0.090 | 0.99 (0.98, 1.00) | 0.084 | 0.99 (0.98, 1.00) | 0.080 |
| Baseline QRS (ms) | 1.01 (0.96, 1.05) | 0.7 | 0.97 (0.92, 1.01) | 0.2 | 0.97 (0.92, 1.01) | 0.2 | 0.98 (0.94, 1.02) | 0.3 | 0.97 (0.92, 1.01) | 0.2 | 0.97 (0.92, 1.01) | 0.2 |
| B-blockers | 0.01 (0.00, 0.21) | 0.003 | 0.01 (0.00, 0.19) | 0.002 | 0.01 (0.00, 0.19) | 0.002 | 0.02 (0.00, 0.21) | 0.002 | 0.01 (0.00, 0.20) | 0.002 | 0.01 (0.00, 0.19) | 0.002 |

Abbreviations: CRT, cardiac resynchronization therapy; LBBB, left bundle branch block; aHR, adjusted hazard ratio; CI, confidence interval; HF, heart failure; AF, atrial fibrillation; NYHA, New York Heart Association; LVEF, left ventricular ejection fraction; LVESV, left-ventricular end-systolic volume; ESC, European Society of Cardiology; WHO, World Health Organization; AHA, American Heart Association.

**Table S.8:** Multivariable negative binomial regression for hospitalization rates after CRT by different criteria of left bundle branch block (LBBB)

| **Variable** | **LBBB Strauss** | | **LBBB Marriott** | | **LBBB Perrin** | | **LBBB ESC 2013** | | **LBBB ESC 2021** | | **LBBB WHO/AHA** | |
| --- | --- | --- | --- | --- | --- | --- | --- | --- | --- | --- | --- | --- |
|  | **aIRR (95% CI)** | **p-value** | **aIRR (95% CI)** | **p-value** | **aIRR (95% CI)** | **p-value** | **aIRR (95% CI)** | **p-value** | **aIRR (95% CI)** | **p-value** | **aIRR (95% CI)** | **p-value** |
| LBBB | 0.30 (0.13, 0.64) | 0.002 | 0.48 (0.21, 1.05) | 0.071 | 0.44 (0.19, 0.96) | 0.045 | 1.09 (0.48, 2.54) | 0.8 | 0.51 (0.23, 1.09) | 0.093 | 0.43 (0.18, 0.94) | 0.041 |
| Age (years) | 0.97 (0.93, 1.00) | 0.060 | 0.96 (0.92, 0.99) | 0.014 | 0.96 (0.92, 0.99) | 0.015 | 0.96 (0.93, 1.00) | 0.021 | 0.96 (0.92, 0.99) | 0.011 | 0.96 (0.92, 0.99) | 0.013 |
| Sex (Male vs Female) | 0.79 (0.31, 2.05) | 0.6 | 0.93 (0.37, 2.38) | 0.9 | 0.91 (0.36, 2.35) | 0.8 | 1.09 (0.44, 2.73) | 0.9 | 0.93 (0.37, 2.40) | 0.9 | 0.87 (0.34, 2.27) | 0.8 |
| HF type (Non-ischemic vs Ischemic) | 0.58 (0.29, 1.16) | 0.12 | 0.51 (0.25, 1.03) | 0.055 | 0.50 (0.25, 1.01) | 0.048 | 0.48 (0.24, 0.93) | 0.029 | 0.49 (0.24, 0.97) | 0.038 | 0.51 (0.25, 1.02) | 0.052 |
| Paroxysmal AF | 1.55 (0.80, 3.03) | 0.2 | 1.54 (0.79, 3.03) | 0.2 | 1.52 (0.78, 3.00) | 0.2 | 1.45 (0.72, 2.92) | 0.3 | 1.51 (0.77, 2.98) | 0.2 | 1.56 (0.80, 3.09) | 0.2 |
| NYHA (III/IV vs II) | 1.88 (0.69, 5.70) | 0.2 | 1.79 (0.65, 5.46) | 0.3 | 1.60 (0.58, 4.90) | 0.4 | 1.72 (0.64, 5.10) | 0.3 | 1.63 (0.59, 4.92) | 0.4 | 1.81 (0.65, 5.55) | 0.3 |
| Baseline QRS (ms) | 0.99 (0.97, 1.01) | 0.4 | 0.98 (0.97, 1.00) | 0.11 | 0.99 (0.97, 1.00) | 0.12 | 0.98 (0.96, 1.00) | 0.017 | 0.98 (0.97, 1.00) | 0.090 | 0.99 (0.97, 1.00) | 0.14 |
| LVEF (%) | 1.00 (0.92, 1.08) | >0.9 | 0.99 (0.91, 1.07) | 0.8 | 0.99 (0.91, 1.07) | 0.7 | 0.97 (0.90, 1.05) | 0.4 | 0.99 (0.91, 1.07) | 0.8 | 0.99 (0.91, 1.07) | 0.8 |
| LVESV (ml) | 1.00 (0.99, 1.01) | >0.9 | 1.00 (1.0, 1.01) | 0.8 | 1.00 (0.99, 1.01) | 0.8 | 1.00 (0.99, 1.01) | >0.9 | 1.00 (0.99, 1.01) | 0.9 | 1.00 (1.0, 1.01) | 0.8 |
| MRA | 2.93 (0.93, 11.9) | 0.084 | 2.51 (0.80, 9.72) | 0.13 | 2.53 (0.80, 9.88) | 0.12 | 2.28 (0.75, 8.55) | 0.2 | 2.43 (0.78, 9.36) | 0.14 | 2.55 (0.81, 9.99) | 0.12 |
| Antiarrhythmics | 2.09 (1.07, 4.15) | 0.034 | 2.46 (1.21, 5.12) | 0.014 | 2.56 (1.25, 5.42) | 0.011 | 2.08 (1.06, 4.14) | 0.038 | 2.40 (1.18, 4.99) | 0.017 | 2.50 (1.23, 5.23) | 0.013 |

Abbreviations: CRT, cardiac resynchronization therapy; LBBB, left bundle branch block; aIRR, adjusted incidence rate ratio; HF, heart failure; AF, atrial fibrillation; NYHA, New York Heart Association; EF, ejection fraction; LVESV, left-ventricular end-systolic volume; MRA, mineralocorticoid receptor antagonists; ESC, European Society of Cardiology; WHO, World Health Organization; AHA, American Heart Association.

**Supplementary References**

1. Daubert JC, Saxon L, Adamson PB, Auricchio A, Berger RD, Beshai JF, et al. 2012 EHRA/HRS expert consensus statement on cardiac resynchronization therapy in heart failure: implant and follow-up recommendations and management. Heart Rhythm 2012;9:1524–1576.

2. Linde C, Abraham WT, Gold MR, Daubert JC, Tang AS, Young JB, et al. Predictors of short-term clinical response to cardiac resynchronization therapy. Eur J Heart Fail 2017;19:1056–1063.

3. Martins R, António N, Donato H, Oliveiros B. Predictors of echocardiographic response to cardiac resynchronization therapy: a systematic review with meta-analysis. IJC Heart Vasc 2022;39:100979.

4. Galloo X, Khidir M, Stassen J, Hirasawa K, Cosyns B, van der Bijl P, et al. Risk factors for short-term versus long-term mortality in patients who underwent cardiac resynchronization therapy. Am J Cardiol 2023;197:34–41.
